# Supplementary material for: Nicotinamide Riboside Supplementation to Suckling Male Mice Improves Lipid and Energy Metabolism in Skeletal Muscle and Liver in Adulthood
Source: Nutrients. 2022 May 28;14(11):2259. doi: 10.3390/nu14112259 (PMC9182637; doi:10.3390/nu14112259)
Supplement: Supplementary file 1 [file nutrients-14-02259-s001.zip › nutrients-1720669-supplementary.pdf]

Supplementary material: Nicotinamide riboside supplementation to suckling male mice improves lipid and energy metabolism in skeletal muscle and liver in adulthood by Alba Serrano, Andreu Palou, M. Luisa Bonet, Joan Ribot

Supplementary Table S1. Early-life nicotinamide riboside (NR) treatment attenuated high-fat (HF) diet-induced body fat accumulation and energy efficiency in adulthood despite higher food intake.

	control		NR		
	NF	HF	NF	HF	ANOVA
Body weight (g)	44.6 ± 1.2	52.7 ± 2.6#	45.0 ± 1.9	52.3 ± 1.9#	D
Inguinal WAT (g)	0.685 ± 0.092	1.10 ± 0.13#	0.672 ± 0.044	1.04 ± 0.12	D
Epididymal WAT (g)	1.30 ± 0.22	2.08 ± 0.23#	1.27 ± 0.16	2.13 ± 0.43	D
Retroperitoneal WAT (g)	0.295 ± 0.037	0.506 ± 0.053#	0.328 ± 0.041	0.485 ± 0.095	D
Adipose index (%)	5.04 ± 0.59	7.03 ± 0.61#	5.00 ± 0.35	6.82 ± 1.18	D
Energy intake (kcal/mice)	1,176 ± 44	1,237 ± 36	1,146 ± 34	1,394 ± 73#	D
Energy efficiency (g/Mcal)	0.658 ± 0.357	4.45 ± 0.131#	0.011 ± 1.250	4.93 ± 1.49	D

Newborn male mice received NR or vehicle (water; control) from day 2 to 20 of age, were weaned onto a chow diet on day 21, and were assigned to either a HF or a normal-fat (NF) diet on day 90, for 10 weeks. Body and white adipose tissue (WAT) pad weights, and adiposity index (combined mass of all fat pads taken expressed as a percentage of body weight) at the end of the experiment, data are the mean±SEM of n=5-6 animals per group. Cumulative food intake and energy efficiency (body weight gained per calories consumed) are calculated on a per-cage basis and are the mean±SEM of 2-3 cages per group (2-3 animals per cage) along with HF diet challenge. Statistics ($P<0.05$): D diet effect (two-way ANOVA); # HF vs NF (t-Student).

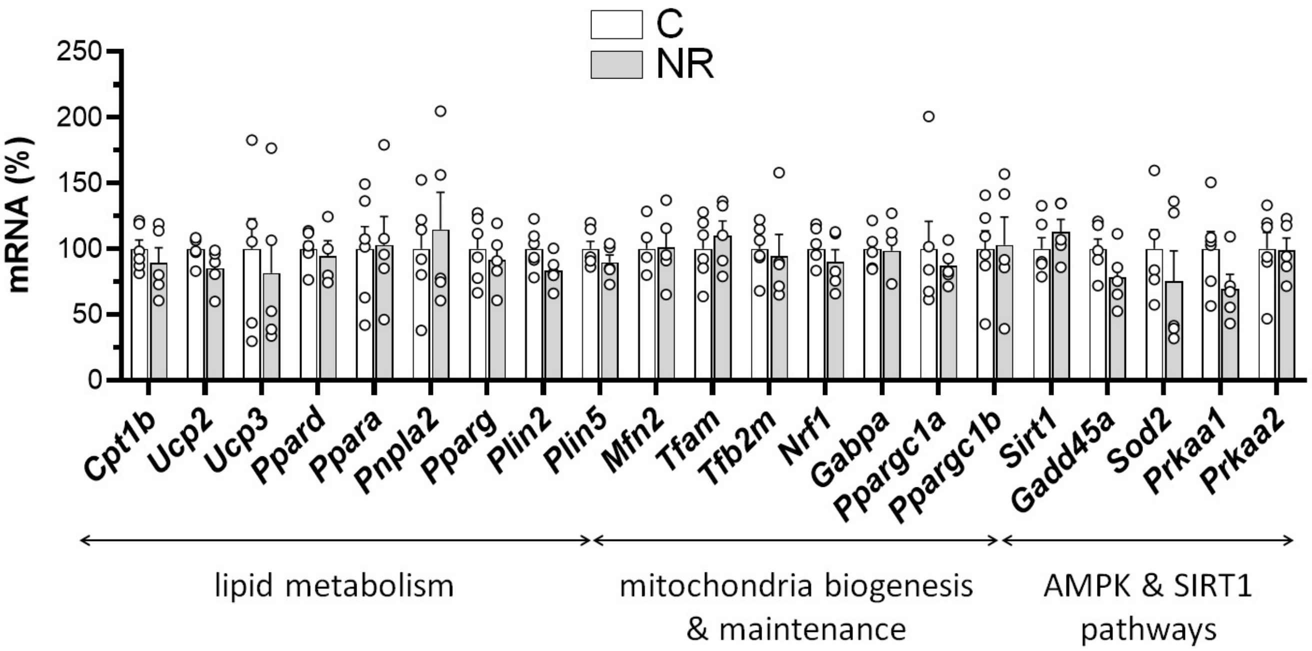

Supplementary Figure S1. Early-life nicotinamide riboside (NR) treatment did not significantly affect lipid metabolism capacities and mitochondria pathways in skeletal muscle of mice at a young age. Newborn male mice received NR or vehicle (water; control) from day 2 to 20 of age and were weaned onto a chow diet on day 21. mRNA levels of the indicated genes were analyzed in *Gastrocnemius* skeletal muscle at age of 35 days. Data are the mean±SEM of 5-6 animals per group and are expressed relative to the mean value of the control group, which was set to 100. Circles correspond to individual data.

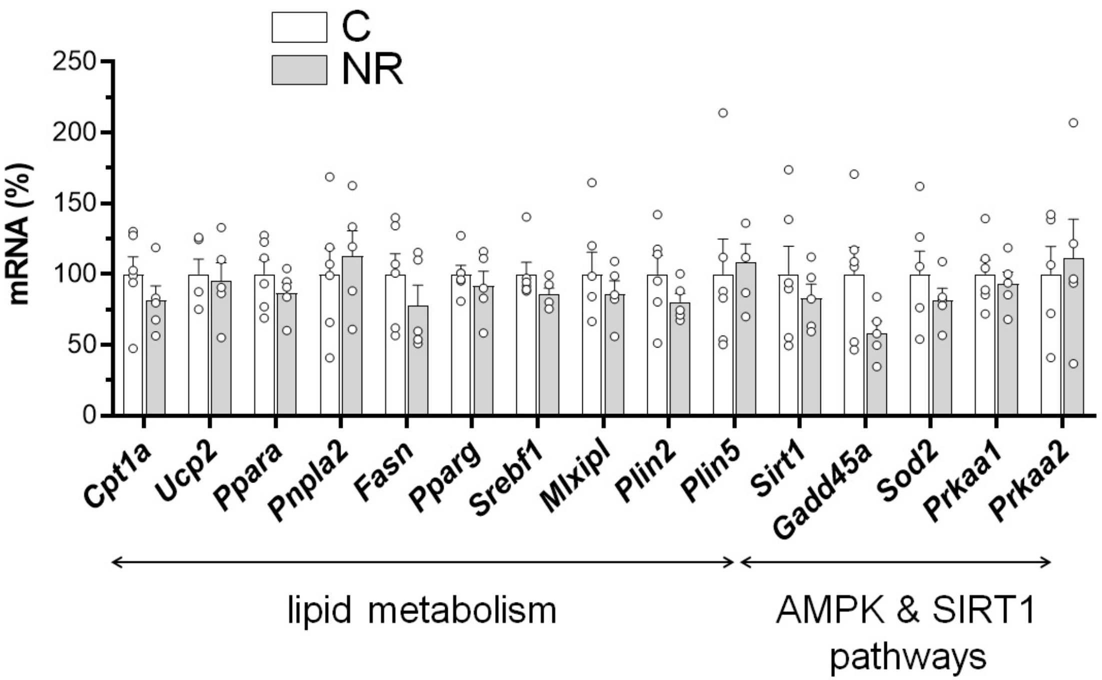

Supplementary Figure S2. Early-life nicotinamide riboside (NR) treatment did not significantly affect lipid metabolism capacities in liver of mice at a young age. Newborn male mice received NR or vehicle (water; control) from day 2 to 20 of age and were weaned onto a chow diet on day 21. mRNA levels of the indicated genes were analyzed in liver at age of 35 days. Data are the mean±SEM of 5-6 animals per group and are expressed relative to the mean value of the control group, which was set to 100. Circles correspond to individual data.

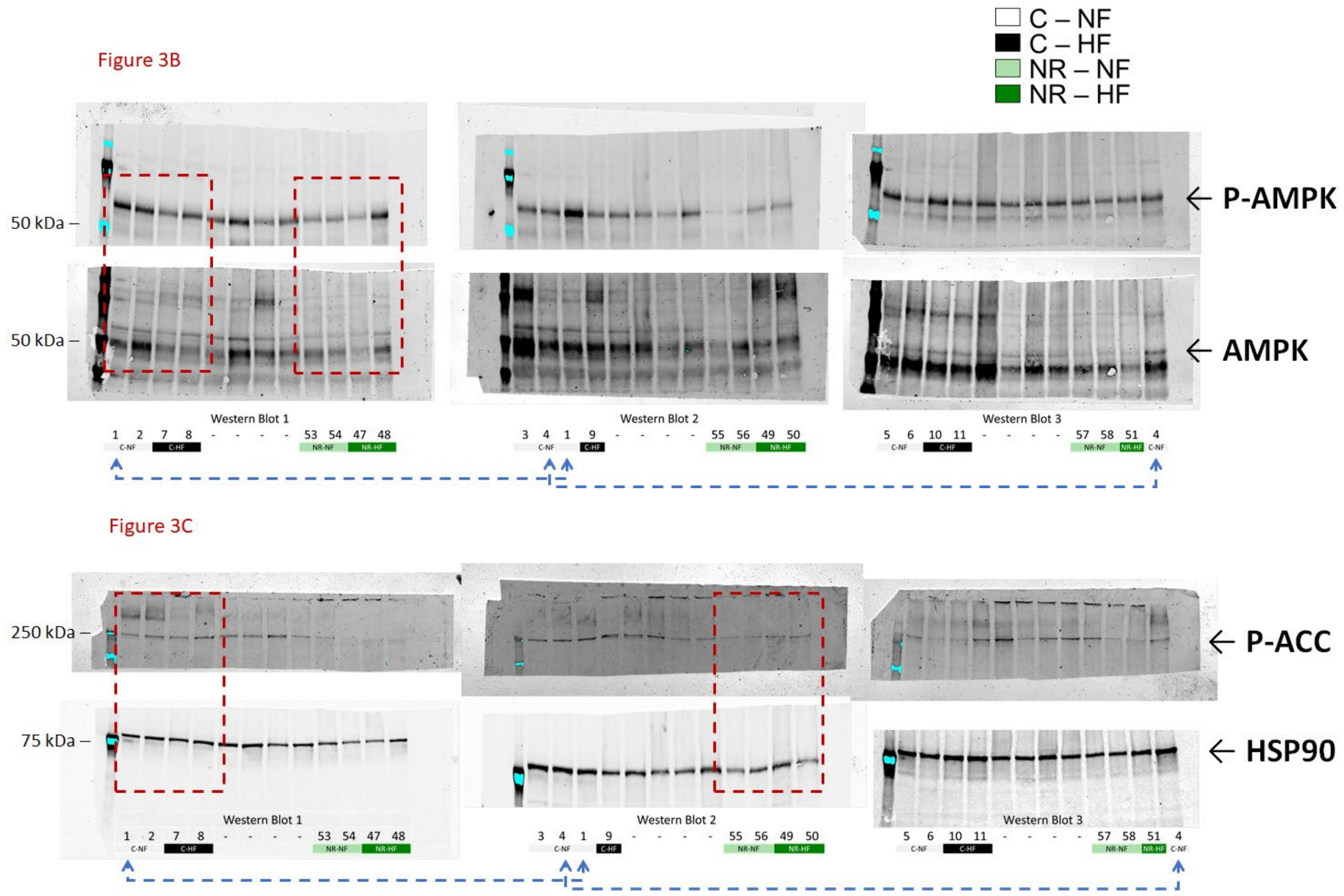

Supplementary Figure S3. Original images supporting western blot results shown in the histograms in Figure 3 panels B and C. All independent biological replicates used in the analysis are included (5-6 animals per group). Red boxes correspond to the cropped images used for illustration in Figure 3. Blue lines connect the technical replicates used to compare membranes with each other.
